# Luminal B, Human Epidermal Growth Factor Receptor 2 (HER2/neu), and Triple-Negative Breast Cancers Associated With a Better Chemotherapy Response Than Luminal A Breast Cancers in Postneoadjuvant Settings

**DOI:** 10.7759/cureus.40066

**Published:** 2023-06-06

**Authors:** Atif A Hashmi, Ummara Bukhari, Javeria Najam, Tanim Dowlah, Abrahim H Ali, Muhammad Asad Diwan, FNU Anjali, Sunder Sham, Shamail Zia, Muhammad Irfan

**Affiliations:** 1 Pathology, Liaquat National Hospital and Medical College, Karachi, PAK; 2 Internal Medicine, Jinnah Sindh Medical University, Karachi, PAK; 3 Internal Medicine, Liaquat National Hospital and Medical College, Karachi, PAK; 4 Internal Medicine, Bangladesh Medical College, Dhaka, BGD; 5 Pathology, The Aga Khan University, Karachi, PAK; 6 Internal Medicine, Sakhi Baba General Hospital, Sukkur, PAK; 7 Pathology, Lenox Hill Hospital, New York, USA; 8 Pathology, Jinnah Sindh Medical University, Karachi, PAK; 9 Statistics, Liaquat National Hospital and Medical College, Karachi, PAK

**Keywords:** chemotherapy response, her2/neu, luminal b, luminal a, postneoadjuvant breast cancer

## Abstract

Background

Breast cancer is a heterogeneous disease with many histological and molecular/intrinsic breast cancer subtypes. Intrinsic breast cancer subtypes include luminal A, luminal B, human epidermal growth factor receptor 2 (HER2/neu), and triple-negative subtypes. The intrinsic breast cancer typing is based on the expression of estrogen receptor (ER), progesterone receptor (PR), HER2/neu, and Ki67-labeling index. One of these patients' foremost prognostic factors upon surgical resection is a response to neoadjuvant chemotherapy. The presence of a pathologically complete response (pCR) indicates a favorable patient outcome compared with a pathologically partial response (pPR). In this study, we compared the neoadjuvant chemotherapy response in breast cancer in different intrinsic breast cancer subtypes.

Methodology

It was a retrospective cross-sectional study conducted in the Department of Histopathology, Liaquat National Hospital, from January 2019 to December 2022, over three years. A total of 287 post-neoadjuvant chemotherapy cases of breast cancer were included. Anthracyclines and taxanes, coupled with or without anti-HER2/neu therapy, have been used in the neoadjuvant chemotherapy treatment setting contingent upon the patients' HER2/neu status. The post-chemotherapy response was assessed pathologically and categorized into pCR and pPR.

Results

The mean age of the patients was 47.90 ± 10.34 years, with a mean tumor size and Ki67 index of 5.36 ± 2.59 cm and 36.30 ± 22.14%, respectively. Invasive breast carcinoma of no special type (IBC-NST) made up 88.2% of cases, while grade 2 carcinomas made up 45.5%. The majority of tumors (42.7%) belonged to tumor (T) stage T2, and nodal metastasis was detected in 59.7% of patients. The intrinsic breast cancer subtypes luminal B (40.6%) and triple negative (33.3%) were the most prevalent, followed by luminal A (15.8%) and HER2/neu (10.3%). In 81 cases (24.5%), pCR was detected. The association of post-neoadjuvant chemotherapy response with intrinsic breast cancer subtypes showed a significant difference (*P *< 0.001). The highest frequency of pCR was noted in HER2/neu cancers (58.8%), followed by luminal B (25.4%) and triple negative (23.6%). Regarding age, T-stage, tumor grade, and histological type of carcinoma, there was no discernible difference between pCR and pPR. Conversely, a significant association was noted for the Ki67 index. A Ki67 index higher than 25% showed a significantly higher frequency of pCR.

Conclusions

In postchemotherapy specimens, the HER2/neu breast cancer subtype substantially displayed higher pCR, followed by luminal B and triple-negative subtypes. After identifying the patients' subtypes, intrinsic subtyping can help determine the prognosis and anticipated response to chemotherapy. Furthermore, prechemotherapy breast specimens with high Ki67 index values have shown a direct association with neoadjuvant chemotherapy response.

## Introduction

Breast cancer is a heterogeneous disease with many histological and molecular/intrinsic breast cancer subtypes. Intrinsic breast cancer subtypes include luminal A, luminal B, human epidermal growth factor receptor 2 (HER2/neu), and triple-negative breast cancer (TNBC) subtypes. Intrinsic breast cancer typing is based on the expression of estrogen receptor (ER), progesterone receptor (PR), HER2/neu, and Ki67 labeling index [1,2].

According to statistics, Pakistan has one of the highest rates of breast cancer [3]. This is owing to the paucity of resources in this part of the world and the significant number of patients arriving at advanced stages of illness, making early surgery impractical. In these situations, neoadjuvant chemotherapy is used to decrease the severity of the disease before it is surgically removed. In contrast, in Western countries, particularly at early stages, exceptionally efficient detection programs such as mammography have rendered it achievable to recognize this cancer early and have swift curative surgery.

Upfront surgery is generally indicated in early breast cancers. In contrast, neoadjuvant chemotherapy followed by surgery is the most commonly used management option in advanced breast cancers. In Pakistan, most of the patients present at an advanced stage; therefore, many cases undergo postneoadjuvant chemotherapy [4].

One of these patients' foremost prognostic factors upon surgical resection is the continued existence of remnant tumor cells in the surgically excised specimens, which is employed for foreseeing pathogenic reactions. The presence of a pathologically complete response (pCR) indicates a favorable patient outcome compared with a pathologically partial response (pPR) [5]. In this study, we compared the neoadjuvant chemotherapy response in breast cancer in different intrinsic breast cancer subtypes. This study will help construct statistics because there is a paucity of data available regarding it in our population as a whole. It will help determine whether particular subtypes have a greater likelihood to respond more favorably to neoadjuvant chemotherapy, which might impact a patient's prognosis upon initial contact.

## Materials and methods

It was a retrospective cross-sectional study conducted in the Department of Histopathology, Liaquat National Hospital and Medical College, from January 2019 to December 2022, for three years. Breast cancers that were treated at our institute were included in the study. Only those cases that had neoadjuvant chemotherapy before surgery were included, while those with upfront surgery were excluded from the analysis. The cases with systemic metastasis at the time of presentation along with male breast cancer cases were also excluded from the study. Clinical data were obtained from hospital records, while histopathology records were obtained from department archives. Cases with incomplete clinical or pathological records were excluded from the analysis, along with cases in which biomarker studies were not conducted.

A Trucut biopsy was performed on all cases to confirm breast cancer. A histological diagnosis of breast cancer was performed, and the tumor type and grade were determined. Immunohistochemical (IHC) studies for ER, PR, HER2/neu, and Ki67 were performed on Trucut biopsy material to determine intrinsic breast cancer subtype. Fluorescence in situ hybridization (FISH) was performed in cases with equivocal IHC (2+) results for HER2/neu. Amplified FISH results were taken as HER2/neu positive. For Ki67 IHC, nuclear staining was interpreted. The number of positively stained cancer cells was counted in at least 10 high-power fields (HPFs), taking into account the hot spots, and an average percentage was calculated.

Breast cancer subtyping was performed according to the criteria shown in Table 1.

**Table 1:** Criteria for intrinsic breast cancer subtyping. ER, estrogen receptor; PR, progesterone receptor; HER2/neu, human epidermal growth factor receptor 2

Intrinsic breast cancer subtype	Criteria
Luminal A	ER-positive or PR-positive (>20%), HER2/neu-negative, and Ki67 <14%
Luminal B	ER-positive or PR-positive (any percentage), HER2/neu-positive, and any Ki67; or ER-positive, PR-positive (<20%), HER2/neu-negative, and any Ki67; or ER-positive, PR-positive (any percentage), HER2/neu-negative, and Ki67 >14%
HER2/neu	ER-negative, PR-negative, HER2/neu-positive, and any Ki67
Triple-negative	ER-negative, PR-negative, HER2/neu-negative, and any Ki67

All patients received neoadjuvant chemotherapy before surgery. Anthracyclines and taxanes, coupled with or without anti-HER2/neu therapy (Herceptin), have been used in the neoadjuvant chemotherapy treatment setting contingent upon the patients' HER2/neu status. After neoadjuvant chemotherapy, surgical excision was performed depending upon the radiological assessment of residual tumor size. Breast conservation surgery/lumpectomy was performed in small tumors, whereas cases with large tumors underwent mastectomy or modified radical mastectomy. The axillary lymph node dissection was performed depending on the status of the sentinel lymph nodes. Sentinel lymph nodes were assessed on the frozen section. Cases with positive sentinel lymph nodes on the frozen section underwent an axillary dissection.

Postchemotherapy specimens were received in the histopathology laboratory. Tumor bed size was estimated grossly and recorded, and mapped representative sections were taken from the tumor bed to assess residual tumor size microscopically. Surgical margins were also recorded. A microscopic examination was performed to accurately determine residual tumor size. The postchemotherapy response was categorized according to the criteria shown in Table 2.

**Table 2:** Criteria for postneoadjuvant chemotherapy response categorization in breast cancer. pCR, pathologically complete response; pPR, pathologically partial response

Postneoadjuvant chemotherapy response	Criteria
Complete chemotherapy response (pCR)	No residual cancer cells in the breast or axillary lymph nodes after extensive sampling of the tumor bed
Partial chemotherapy response (pPR)	Residual cancer cells present in breast or axillary lymph nodes with appreciable chemotherapy response (fibrosis, histiocytic infiltration, and infarction)
No chemotherapy response	No significant reduction in tumor cells, no appreciable chemotherapy response present (fibrosis, histiocytic infiltration, and infarction)

Data analysis was performed using the Statistical Package for Social Sciences (Version 26.0, IBM Corp., Armonk, NY, USA). The chi-square test, independent t-test, and Fisher’s exact test were used to check the association. *P*-values <0.05 were considered significant.

## Results

There were 287 patients in the study. The demographics and clinical characteristics of the study population are shown in Table 3. The mean age of the patients was 47.90 ± 10.34 years, with a mean tumor size and Ki67 index of 5.36 ± 2.59 cm and 36.30 ± 22.14%, respectively. Invasive breast carcinoma of no special type (IBC-NST) was the most common histological subtype comprising 88.2% of cases, while grade 2 was the most common prechemotherapy tumor grade (45.5%). The majority of tumors (42.7%) belonged to tumor (T) stage T2, and nodal metastasis was detected in 59.7% of patients. The intrinsic breast cancer subtypes luminal B (40.6%) and triple-negative breast cancer (TNBC, 33.3%) were the most prevalent, followed by luminal A (15.8%) and Her2/neu (10.3%). In 81 cases (24.5%), pCR was detected, while pPR was detected in 75.5% of cases. In our study, none of the cases had *no chemotherapy response*.

**Table 3:** Clinicopathological profile of the population under study. SD, standard deviation; NST, no special type; MRM, modified radical mastectomy; T, tumor; N, nodal; HER2/neu, human epidermal growth factor receptor 2 protein

Clinicopathological features	Values
Age (years), mean ± SD	47.90 ± 10.34
Tumor size (cm), mean ± SD	5.36 ± 2.59
Ki67 (%), mean ± SD	36.30 ± 22.14
Histological subtype	
Invasive breast carcinoma, NST, *n* (%)	291 (88.2)
Others, *n* (%)	39 (11.8)
Intrinsic breast cancer subtypes	
Luminal A, *n* (%)	52 (15.8)
Luminal B, *n* (%)	134 (40.6)
HER2/neu, *n* (%)	34 (10.3)
Triple negative, *n* (%)	110 (33.3)
Nodal metastasis	
Present, *n* (%)	197 (59.7)
Absent, *n* (%)	133 (40.3)
Type of surgery	
Breast conservation surgery, *n* (%)	115 (34.8)
MRM, *n* (%)	140 (42.4)
Simple mastectomy, *n* (%)	75 (22.7)
Laterality	
Right, *n* (%)	178 (53.9)
Left, *n* (%)	152 (46.1)
Tumor grade	
Grade 1, *n* (%)	57 (17.3)
Grade 2, *n* (%)	150 (45.5)
Grade 3, *n* (%)	123 (37.3)
T stage	
T0, n (%)	4 (1.2)
T1, *n* (%)	37 (11.2)
T2, *n* (%)	141 (42.7)
T3, *n* (%)	68 (20.6)
T4, *n* (%)	80 (24.2)
N stage	
N0, *n* (%)	133 (40.3)
N1, *n* (%)	152 (46.1)
N2, *n* (%)	42 (12.7)
N3, *n* (%)	3 (0.9)
Chemotherapy response	
Partial response, *n* (%)	249 (75.5)
Complete response, *n* (%)	81 (24.5)

**Figure 1:**
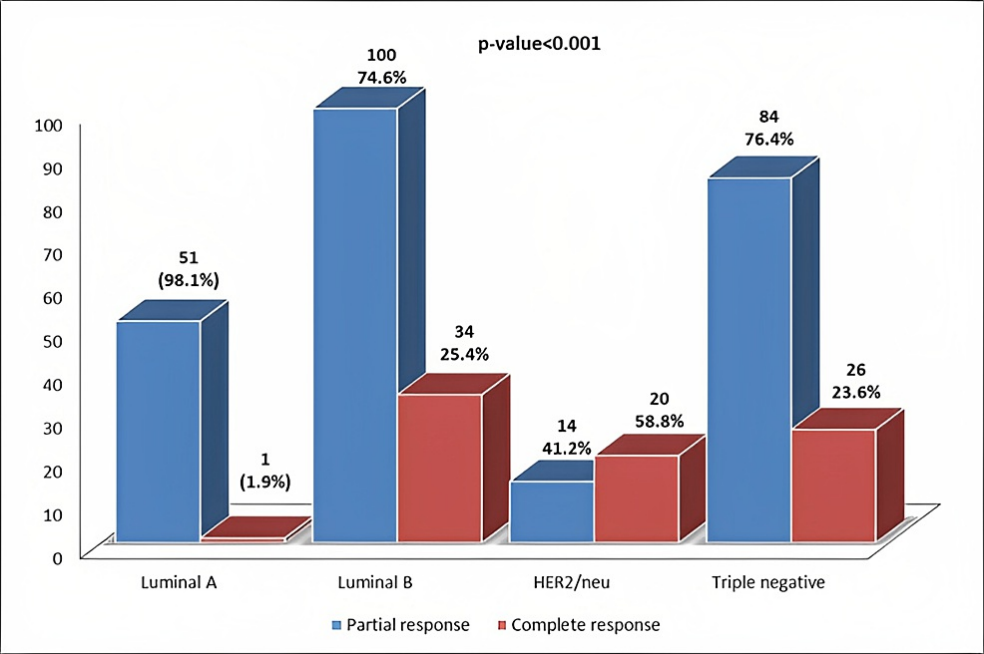
Association of postneoadjuvant chemotherapy response with intrinsic breast cancer subtypes. HER2/neu, human epidermal growth factor receptor 2 protein

The association of postneoadjuvant chemotherapy response with intrinsic breast cancer subtypes is depicted in Figure 1. A significant difference in chemotherapy response was noted among intrinsic breast cancer subtypes (*P *< 0.001). The highest frequency of pCR was noted in HER2/neu cancers (58.8%), followed by luminal B (25.4%) and TNBC (23.6%).

Table 4 displays the association between clinicopathological parameters and neoadjuvant therapy response. Regarding age, T-stage, tumor grade, and histological type of carcinoma, there was no discernible difference between pCR and pPR. Conversely, a significant association was noted for the Ki67 index and nodal (N) stage. A higher mean Ki67 index was noted in the pCR group. Similarly, the N0 and N2 stages showed a slightly higher frequency of pCR.

**Table 4:** Association of postneoadjuvant chemotherapy response in breast cancer with clinicopathological parameters. SD, standard deviation; NST, no special type; T, tumor; N, nodal *Independent t-test was applied. **P-value < 0.05 was significant. ***Chi-square test was applied. ****Fisher’s exact test was applied.

Clinicopathological parameters	Values		*P*-value
	Postneoadjuvant chemotherapy response		
	Partial response	Complete response	
Age (years), mean ± SD*	47.75 ± 10.49	48.37 ± 9.90	0.645
Tumor size (cm), mean ± SD*	5.30 ± 2.58	5.55 ± 2.62	0.440
Ki67 index (%), mean ± SD*	34.81 ± 22.21	40.87 ± 21.42	0.032**
Histological subtype***			
Invasive breast carcinoma, NST, *n* (%)	221 (88.8)	70 (86.4)	0.572
Others, *n* (%)	28 (11.2)	11 (13.6)	
Nodal metastasis***			
Present, *n* (%)	150 (60.2)	47 (58)	0.724
Absent, *n* (%)	99 (39.8)	34 (42)	
Tumor grade***			
Grade 1, *n* (%)	45 (18.1)	12 (14.8)	0.771
Grade 2, *n* (%)	113 (45.4)	37 (45.7)	
Grade 3, *n* (%)	91 (36.5)	32 (39.5)	
T stage****			
T0, *n* (%)	4 (1.6)	0 (0)	0.503
T1, *n* (%)	27 (10.8)	10 (12.3)	
T2, *n* (%)	103 (41.4)	38 (46.9)	
T3, *n* (%)	56 (22.5)	12 (14.8)	
T4, *n* (%)	59 (23.7)	21 (25.9)	
N stage****			
N0, *n* (%)	99 (39.8)	34 (42)	0.042**
N1, *n* (%)	122 (49)	30 (37)	
N2, *n* (%)	25 (10)	17 (21)	
N3, *n* (%)	3 (1.2)	0 (0)	

**Figure 2:**
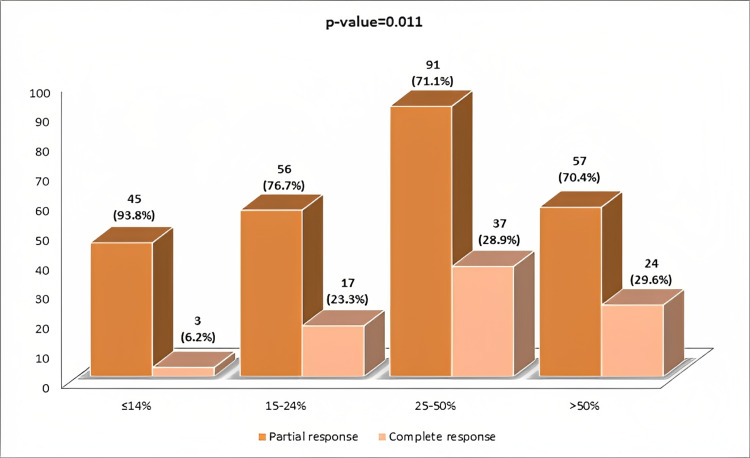
Association of postneoadjuvant chemotherapy response in breast cancer with the Ki67 proliferation index.

Figure 2 shows the association of postneoadjuvant chemotherapy response with Ki67 index groups. A Ki67 index higher than 25% showed a significantly higher frequency of pCR.

## Discussion

In this study, we found that following neoadjuvant therapy for breast cancer, the HER2/neu breast cancer subtype followed by luminal B and TNBCs demonstrated the highest frequency of pCR. Moreover, the Ki67 proliferation index was also an important factor influencing the response to chemotherapy.

Many international studies have looked at subtypes and other pathological variables that can predict how breast cancer will respond to neoadjuvant chemotherapy. The choice to administer adjuvant systemic therapy to patients with breast cancer is currently made mostly based on histopathological factors such as tumor size, nodal status, grade, and the expression of ER, PR, and HER2/neu. These standards have been proposed in both national and international guidelines [6,7]. One of the most important parameters is the stage of the initial presentation. Patients presenting at an initial stage (T1/T2) may benefit from upfront surgery. Unfortunately, in underdeveloped countries like Pakistan, most breast cancer patients present at a later stage (T3/T4). Most centers in Pakistan now offer postneoadjuvant chemotherapy to downstage the disease before surgery.

Many researchers have looked into the frequency of pCR in different intrinsic breast cancer subtypes. In the study involving 211 cases of breast cancer, Omair et al. [8] found a higher proportion of pCR in HER2/neu (45.2%) and TNBCs (28%). These results were consistent with our findings. They also found that pCR was associated with better disease-free and overall survival. Similarly, in the study evaluating postneoadjuvant chemotherapy response in breast cancer, Goldstein et al. [9] found a relatively low frequency of pCR in luminal breast cancers. They also noted that the main reason for this finding was that most lobular breast cancers are luminal that rarely achieve pCR. In contrast, we did not find any significant difference in pCR for the histological subtype.

We also found that tumors with a high Ki67 proliferation index exhibited a higher proportion of pCR. Several research questions are still unanswered, despite being aware that the scientific community is closer than ever to embracing Ki67 as a prognostic and predictive biomarker in the medical care and therapy of breast cancer patients [10]. Ki67 and other breast cancer biomarker assessments are going to be more time-effective because of the implementation of automated and digital scoring techniques that will keep incorporating these latest innovations into the pathology laboratory. The correlation of clinical outcomes with Ki67 as analyzed by gene expression may result in a more accurate description of Ki67 thresholds for prognosis and predictive therapy, corresponding to the validation of HER2 IHC results with FISH [11].

We found that luminal B breast cancers were of the highest frequency in our population, followed by TNBCs. Studies have shown that compared with white women, native African women, and African Americans had a higher prevalence of TNBC [12]. These cancers have poor biological behavior [13]. Conversely, as noted in our study, TNBCs showed a better response to postneoadjuvant chemotherapy than luminal A breast cancers.

Limitations

Our study had a few limitations. First, it was a retrospective study, and clinical follow-up of patients was not performed to evaluate disease-free and overall survival in patients with pCR. Second, the association of some newly emerged biomarkers like programmed death ligand 1 (PD-L1) with pCR was not sought in our study. Moreover, it was a single-center study and so the sample size was limited.

## Conclusions

In our study, we found a low frequency of pCR in postneoadjuvant breast cancer patients. In a comparative analysis, we noted that HER2/neu, followed by luminal B and triple-negative breast cancers, showed the highest frequency of pCR in our study. We also noted that a high Ki67 proliferation index was significantly associated with a higher frequency of pCR. Therefore, we recommend that apart from hormone receptors and HER2/neu, Ki67 should be evaluated in all breast cancers undergoing neoadjuvant chemotherapy to stratify patients who can benefit more from neoadjuvant chemotherapy.
